# High levels of baseline serum IL-10 are associated with reduced clinical benefit from first-line immune checkpoint inhibitor therapy in advanced renal cell carcinoma

**DOI:** 10.7150/jca.81384

**Published:** 2023-04-02

**Authors:** Youngun Kim, Hannah Yang, Won Suk Lee, Jaekyung Cheon, Yun Beom Sang, Beodeul Kang, Hong Jae Chon, Chan Kim

**Affiliations:** 1 CHA University School of Medicine, Seongnam, Korea; 2 Medical Oncology, Department of Internal Medicine, CHA Bundang Medical Center, CHA University School of Medicine, Seongnam, Korea

**Keywords:** biomarkers, immune checkpoint inhibitor, interleukin-10, advanced renal cell carcinoma, survival

## Abstract

Immune checkpoint inhibitor (ICI) became a standard treatment for advanced renal cell carcinoma (RCC). However, clinically valid biomarkers of therapeutic outcome are lacking. We investigated the role of interleukin-10 (IL-10) as a predictive biomarker for first-line ICI therapy in patients with advanced RCC. Baseline serum samples were prospectively collected and analyzed using a cytometric bead assay. Patients were divided into two groups according to their serum IL-10 levels using maximally selected rank statistics. A fraction (13.0%) of patients had high levels of serum IL-10 at baseline. High serum IL-10 levels (> 4.3 ng/mL) were associated with a significantly shorter progression-free (median: 5.2 months vs. not reached, P = 0.007) and overall survival (median: 13.9 months vs. not reached, P < 0.001). Multivariate Cox regression analysis confirmed the independent association between high serum IL-10 levels and poor survival outcomes. Effector cytokine production and the proliferative response of CD8^+^ T cells were significantly lower in patients with high serum IL-10 levels, who also had a shorter duration of response to first-line ICI therapy (4.6 months vs. not reached, P < 0.001). In conclusion, elevated serum IL-10 levels at baseline were associated with reduced clinical benefit from first-line ICI therapy in patients with advanced RCC.

## Introduction

The treatment of advanced renal cell carcinoma (RCC) continues to develop based on a growing understanding of the pathophysiology of the disease 1. Clear cell carcinoma, the most common type of RCC, is characterized by the activation of angiogenic signaling pathways such as vascular endothelial growth factor (VEGF), and hypoxia-inducible factor (HIF) through mutations in the *Von Hippel-Lindau* (*VHL*) gene 2-4. Based on these characteristics, treatments targeting tumor angiogenesis started to emerge in 2006. Sunitinib, an oral multi-targeted receptor tyrosine kinase inhibitor, has a clear survival benefit in patients with advanced RCC compared to interferon alpha 5-7. Since then, various angiogenesis-targeted agents such as pazopanib, bevacizumab, axitinib, cabozantinib, and lenvatinib have been approved for the treatment of advanced RCC 8-15.

The recent emergence of immune checkpoint inhibitors (ICI) has led to a paradigm shift in the landscape of cancer 16-18. RCC is a well-known immunogenic tumor with a high degree of immune cell infiltration that is relatively sensitive to cytokine immunotherapy. It is therefore reasonable to pursue ICI therapy in patients with advanced RCC 17, 19. Nivolumab plus ipilimumab (CheckMate-214 trial) combination therapy has demonstrated a clear survival benefit compared to sunitinib in patient with advanced RCC 20. Later, a combination of anti-angiogenic agents + ICI was investigated. In KEYNOTE-426 trial, pembrolizumab + axitinib showed superior survival outcomes compared to sunitinib 21. Since then, various combinations of ICI and anti-angiogenic agents have been approved as first-line therapy for advanced RCC 22-24.

However, some patients are refractory to ICI treatments. Studies on mechanisms of resistance to ICI therapies are ongoing, but are not yet fully understood 25-28. From this perspective, there is a significant need for clinically viable biomarkers that can predict the clinical response to ICI treatment 29-31.

Interleukin-10 (IL-10) is an immunosuppressive cytokine that inhibits the expression of major histocompatibility complex (MHC) class II molecules and pro-inflammatory cytokines 32. Although the immunological role of IL-10 is relatively well understood, the clinical implications of IL-10 in the tumor microenvironment require further study. Several studies which included patients with melanoma and lung cancer found that high IL-10 levels were correlated with a poor prognosis 33, 34. In RCC, several studies have described IL-10 as a prognostic factor in patients with early stage RCC after surgical resection or in patients with advanced disease treated with cytokine or targeted therapy 35-39. However, the predictive value of IL-10 in patients with advanced RCC treated with first-line ICIs has not been elucidated yet.

Here, we investigated the predictive value of baseline serum IL-10 levels in patients with advanced RCC who received first-line ICIs.

## Method

### Patients and sample preparation

This study prospectively enrolled patients diagnosed with advanced RCC at CHA Bundang Medical Center (Seongnam, Korea) between November 2019 and December 2021. Patients were eligible if they had been pathologically or cytologically diagnosed with RCC, had a target lesion according to Response Evaluation Criteria in Solid Tumors (RECIST) version 1.1, had not received prior systemic therapy, and had an Eastern Cooperative Oncology Group (ECOG) performance status of 0 to 2. Patients with autoimmune disease, immune-compromised conditions, or active infections were excluded from this study. This study was approved by the Institutional Review Board of CHA Bundang Medical Center (CHA 2017-11-054). All patients have written informed consent. Patient age at the time of diagnosis, sex, tumor histology, International Metastatic RCC Database Consortium (IMDC) risk, ECOG performance status, nephrectomy history, metastatic lesion and survival outcomes were obtained from the electronic medical records of CHA Bundang Medical Center. Treatment was continued until disease progression or discontinuation owing to toxicity. Clinical responses were assessed using the RECIST version 1.1. Overall survival (OS) was defined as the duration between the initiation of treatment and the death or the last follow-up. Progression free survival (PFS) was defined as the duration between the initiation of treatment and disease progression or death.

Blood samples was collected before first treatment (baseline) and immediately before the second treatment (cycle 2 day 1, hereafter referred to as C2D1). Serum was diluted 1:2 and cryopreserved at -80℃ in a deep freezer. Peripheral blood mononuclear cells (PBMCs) were isolated from the patient's whole blood using pressure gradient centrifugation with Ficoll-Paque PLUS (GE Healthcare) and cryopreserved.

### Cytometric bead assay (CBA)

Serum IL-10 concentration was measured according to the manual provided by the CBA human Th1/Th2/Th17 Cytokine kit (BD bioscience), as described previously 31. Briefly, the capture beads and detection reagents were incubated with the patient's serum at room temperature for approximately 2 h and 30 min and then washed. Cytokine concentrations were calculated using flow cytometry fluorescence.

### Flow Cytometry analysis

Single-cell suspension of PBMCs were stained using Fixable Viability Dye eFluor^TM^ 780 on ice for 30min to exclude dead cells. The cells were incubated with surface antibodies against CD3 (clone SK7, Invitrogen), CD4 (clone RPA-T4, BioLegend) and CD8 (clone RPA-T8, eBioscience) on ice for 30min, followed by several washes. For intracellular staining, cells were permeabilized using a FoxP3 Fixation and Permeabilization Kit (eBioscience). T cell activation and proliferation assays followed the previous protocols 31, 40. Cells were stained with interferon (IFN)-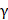 (clone B27, BD Biosciences), tumor necrosis factor (TNF)-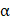 (clone MAB11, BD Biosciences), and Ki-67 (clone Ki-67, BioLegend). Flow Cytometry was performed on a CytoFLEX flow cytometer (Beckman Coulter). The data were analyzed using FlowJo software (Tree Star Inc, Ashland, Oregon, USA).

### Statistical analysis

The IL-10 cut-off value was determined by performing cytokine screening using the maximally selected rank statistics (Maxstat) package of the R software (ver 4.2.2). Statistical analyses were performed using SPSS software version 22. Fisher's exact test and Chi-square test were used to compare the clinical characteristics between the two groups. Kaplan-Meier survival curve analysis and the log-rank test were used to examine the effects of IL-10 on OS and PFS. Multivariate Cox regression analysis was also performed. Statistical significance was set at P < 0.05. All graphs were drawn using GraphPad Prism 8.0 software (GraphPad Software).

## Results

### Patient characteristics

In this study, we analyzed the baseline serum IL-10 levels in 69 patients diagnosed with advanced RCC between November 2019 and December 2021 (Table 1). All the patients were treated with first-line ICI treatment. The median age at the time of treatment initiation was 59 years (range, 19-83), and the majority of the patients were male (n = 49, 71.0%). Most patients had clear cell histology (n = 47, 68.1%). According to the IMDC risk groups, the patients were classified into favorable (n = 15, 21.7%), intermediate (n = 34, 49.3%) and poor risk groups (n = 20, 29.0%). More than half of patients (n = 39, 56.6%) had undergone a previous nephrectomy. The patient cohort had an objective response rate of 47.8% and 1-year OS of 77.9%, with a median follow-up period of 12.1 months.

**Figure 1:**
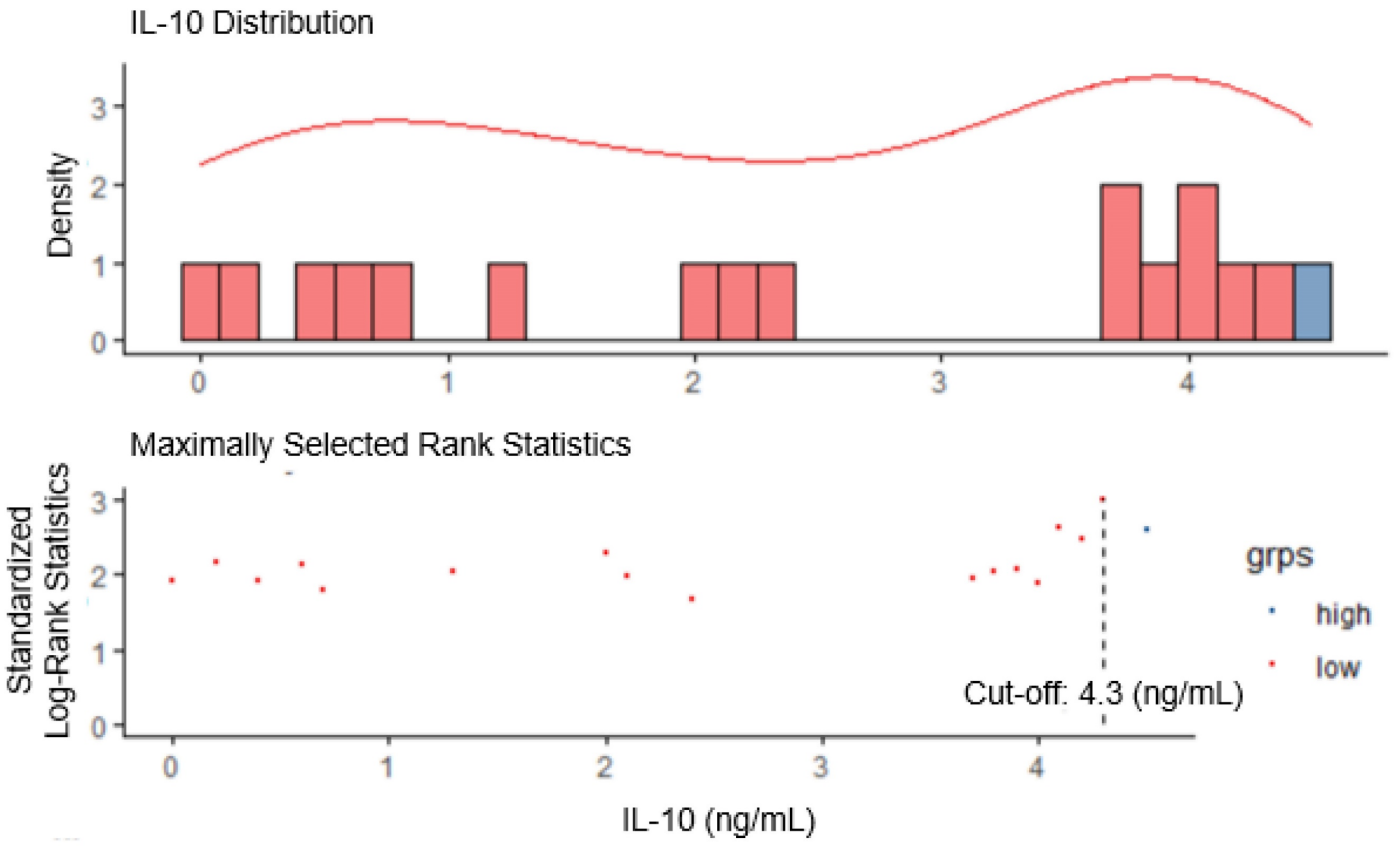
Determination of IL-10 cut-off using maximally selected long-rank statistics in advanced renal cell carcinoma (RCC).

The cut-off value for serum IL-10 was set at 4.3 ng/mL using maximally selected log-rank statistics (Figure 1). Based on this cut-off value, the patients were divided into two groups, i.e., the IL-10-low group (n=60, 87.0%), and the IL-10-high group (n=9, 13.0%). There were no statistically significant differences in the baseline characteristics between IL-10-low and high groups (Table 1). The objective response rate was comparable between two groups (46.6% vs. 55.6%, P = 0.862).

### Elevated baseline serum IL-10 levels were associated with poor survival following ICI therapy in patients with advanced RCC

**Figure 2:**
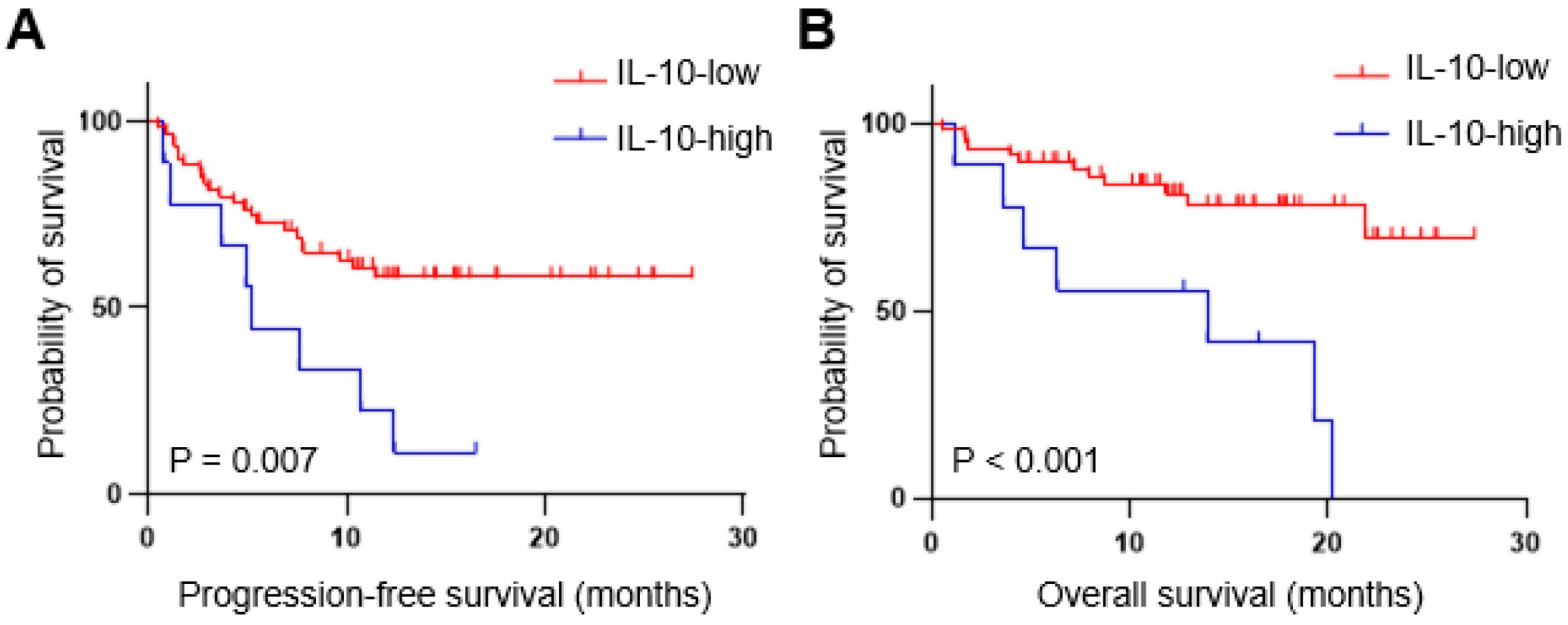
** Patients with high IL-10 levels had unfavorable survival outcomes with first-line immune checkpoint inhibitor (ICI) therapy.** Progression-free (A) and overall (B) survival of the patient with advanced RCC (n=69). IL-10 ≥ 4.3 ng/mL at serum baseline level was defined as IL-10 high. P-values were calculated using the log-rank test. PFS, progression-free survival; OS, overall survival.

Next, we examined the relationship between serum IL-10 levels and clinical outcome (Figure 2). Patients with high IL-10 levels had a significantly shorter median PFS than those with low IL-10 levels (5.2 months vs. not reached, P = 0.007). The median OS was also significantly worse in the IL-10-high vs. those with IL-10-low group (13.9 months vs. not reached, P < 0.001).

**Figure 3:**
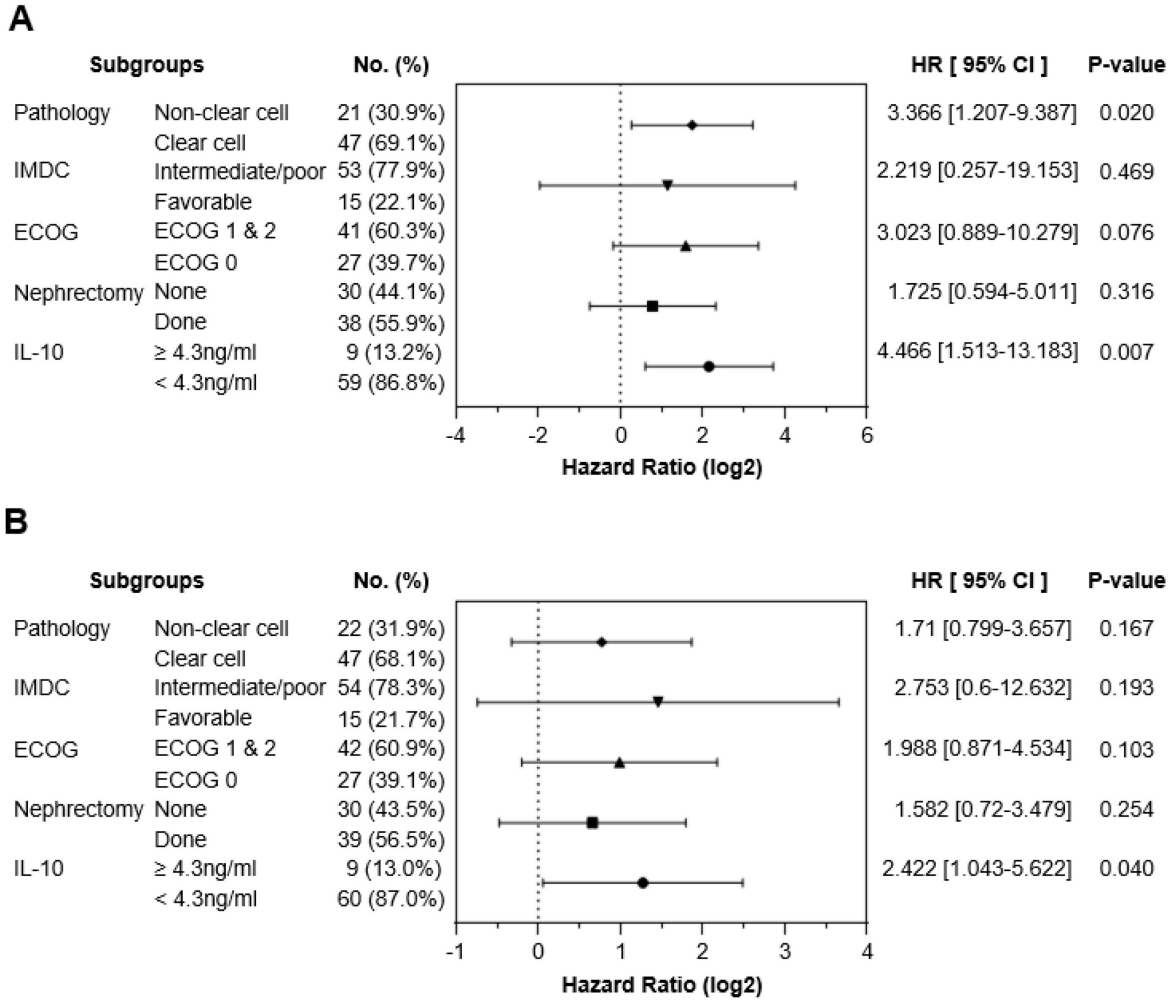
** Serum high IL-10 levels could be an independent predictor for the poor clinical outcome of first-line ICI therapy in advanced RCC.** (A) Forest plot showing multivariable Cox regression analysis data in both OS and PFS according to serum IL-10 levels. Pathology, IMDC risk groups, ECOG stage, and previous history of nephrectomy were adjusted along with serum IL-10 levels as confounding factors. P-value was calculated using the log-rank test. Hazard ratios (HRs) and 95% confidence intervals (CIs) are shown in the plot. IMDC, International Metastatic RCC Database Consortium; ECOG, Eastern Cooperative Oncology Group.

To exclude other factors that could have influenced survival outcomes, a multivariable Cox regression analysis was performed. Potential confounding factors, such as tumor pathology, IMDC risk groups, ECOG performance status, and previous history of nephrectomy, were adjusted for serum IL-10 levels. Multivariate analysis confirmed that the IL-10-high group had poorer OS (Hazard Ratio [HR] 4.466, 95% confidence interval (CI), 1.513-13.183, P = 0.007) and PFS (HR 2.422, 95% CI 1.043-5.622, P = 0.040) compared to the IL-10-low group (Figure 3). These findings suggested that elevated baseline serum IL-10 is an independent predictor of poor clinical outcomes in patients with advanced RCC treated with first-line ICI treatment.

We also analyzed serum IL-10 levels at baseline and C2D1 levels in patients with paired data (n=56). To examine the impact of IL-10 changes between baseline and C2D1, patients were divided into three groups based on the C2D1/Baseline IL-10 ratio: no change or < 1.5 (n = 26), 1.5-2.0 (n = 11), and 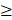 2.0 (n = 15), respectively. The object response rate did not differ significantly among the three groups (57.6% vs. 63.6% vs. 46.7%, respectively, P = 0.673) (Supplementary Table 1). Moreover, neither the PFS (median: not reached vs. 10.7 months vs. 7.8 months, respectively, P = 0.133) nor the OS (median: not reached vs. 19.3 months vs. not reached, respectively, P = 0.229) were statistically different according to the C2D1/baseline IL-10 ratio (Supplementary Figure 1).

### CD8^+^ T-cell function was impaired in the IL-10-high group

**Figure 4:**
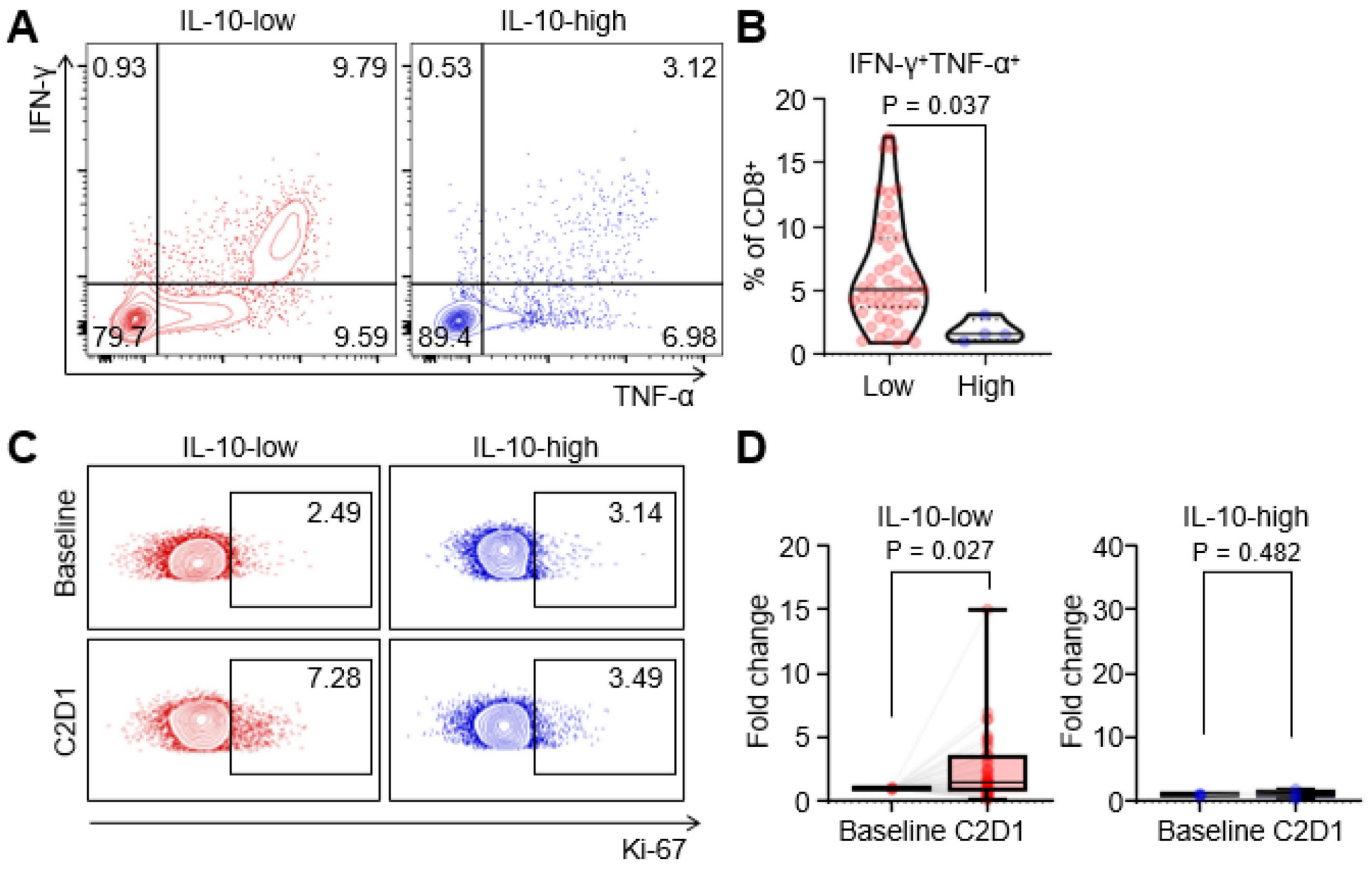
**Impaired T cell function in patients with high IL-10 levels.** (A and B) Representative flow cytometric plot and comparisons of intracellular interferon-γ and tumor necrosis factor-α form CD8^+^ T cells according to IL-10 levels. (C and D) Representative flow cytometric plot and comparisons of Ki-67^+^ proliferating CD8^+^ T cells between baseline and C2D1 according to IL-10 levels. Values were compared using unpaired t-test, with P-values given in each plot.

To elucidate how serum IL-10 levels affect the ICI response, we compared cytokine production and proliferation of T cells between IL-10-high and IL-10-low groups. In the IL-10-high group, the production of IFN-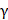 and TNF-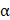 from CD8^+^ T cells was significantly decreased compared to those in the IL-10-low group (Figure 4A, 4B). Furthermore, we compared the fraction of Ki-67^+^ proliferating CD8^+^ T cells before and after the treatment. Proliferative CD8^+^ T cells were remarkably increased at C2D1 in the IL-10-low group, but there were no significant changes in the IL-10-high group (Figure 4C, 4D). Overall, high serum IL-10 levels were associated with impaired cytokines production and decreased CD8^+^ T-cell proliferation.

### The IL-10-high group had a shorter duration of response to first-line ICI therapy

**Figure 5:**
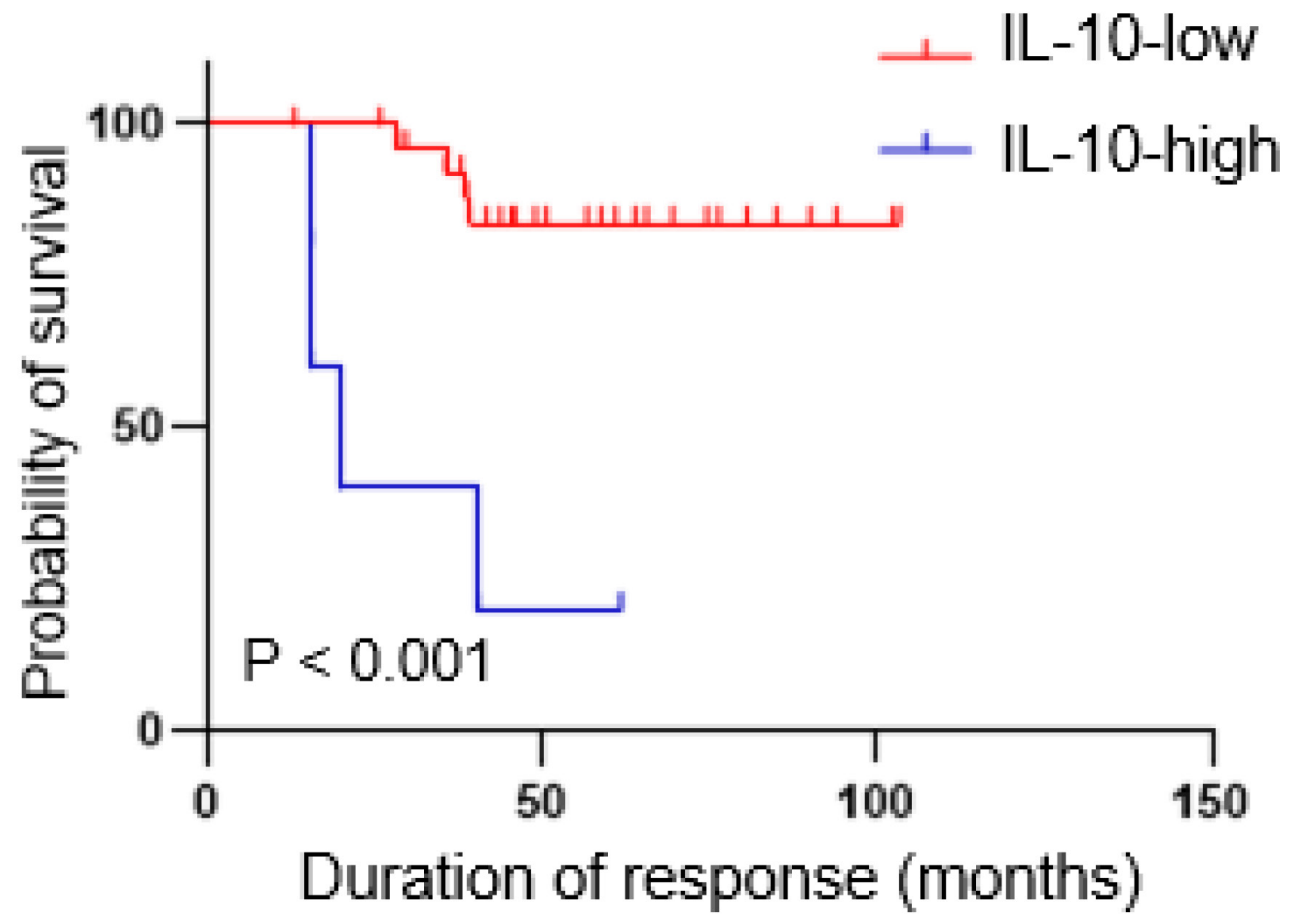
**Patients with high IL-10 levels had shorter duration of response.** Kaplan-Meier curves showing duration of response according to the IL-10 levels. P-values were calculated using the log-rank test.

During follow-up, a continuous response evaluation was performed for all patients, except those with progressive disease (Figure 5). After initial, objective partial or complete responses, most patients in IL-10-low group (n = 24, 85.7%) had durable responses, whereas all but one patient in the IL-10-high group showed a rapid disease progression. Therefore, the median duration of responses was significantly shorter in the IL-10-high vs. the IL-10-low group (4.6 months vs. not reached, P < 0.001).

## Discussion

In this study, we found that patients with advanced RCC and high serum IL-10 levels at baseline had unfavorable survival outcomes when treated with first line ICI therapy. Even after adjusting for other factors that might have influenced clinical outcomes, high baseline serum IL-10 levels were found to be a significant predictor of survival. In addition, high serum IL-10 levels were associated with impaired production of effector cytokines from CD8^+^ T cells and a decreased proliferative response after treatment. Furthermore, even though some patients with high IL-10 levels showed an initial objective response, they exhibited a short duration of responses with rapid progression.

Following the CheckMate-214 and KEYNOTE-426 trials, several combination immunotherapies have been approved by the US Food and Drug Administration for the first-line treatment of advanced RCC 20-24, 41, 42. However, there are no biomarkers capable of predicting which patients with advanced RCC will benefit most from ICI treatment. The present study can help inform the design of future prospective studies towards demonstrating the clinical implications of serum IL-10 levels on survival. Furthermore, our study confirmed the potential of IL-10 as a comprehensive predictive biomarker for immunotherapy by including patient groups with three treatment regimens for advanced RCC.

IL-10 act as an immunosuppressive cytokine in cancer immunity via diverse mechanisms 43-45. Tumor-derived IL-10 induces dendritic cell (DC) dysfunction, resulting in impaired T-cell proliferation and IFN-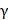 production in murine bladder cancer 46. In addition, macrophage-derived IL-10 suppresses IL-12 secretion from DCs and interferes with CD8^+^ T-cell responses 47. Furthermore, regulatory T-cell-derived IL-10 mediates intratumoral CD8^+^ T-cell exhaustion and restrain anti-tumor immunity 48. In RCC, several studies have suggested prognostic roles of IL-10 in patients with early-stage RCC or in patients with advanced RCC treated with cytokines or targeted therapy. Our study differed from previous studies in that we included only 1) patients with advanced RCC 2) treated with first-line ICI therapy. Furthermore, we suggested IL-10 as a 3) predictive, rather than a prognostic marker, for ICI therapy.

Sullivan et al. recently highlighted IL-10 as a therapeutic target for cancer immunotherapy and demonstrate IL-10-induced T-cell dysfunction can be overcome by IL-10 blockade 49. In that study, anti-IL-10 caused an increase in CD8^+^ T cells without exhaustion gene signatures, and increased the MHC expression in macrophages. Moreover, IL-10 blockade enhances CAR-T cell activation and cytotoxicity, inducing potent tumor cell death in multiple human tumors. Therefore, further studies on how IL-10 mediates immunosuppression in RCC and how it can be overcome through IL-10 blockade are warranted to improve the clinical outcomes in patients with high IL-10 levels.

Our study had several limitations. First, the research was conducted at a single center with a limited number of patients and lacked external validation. Using maximally selected log-rank statistics, we selected 4.3 ng/mL as the cut-off value for IL-10 to maximized statistical significance and minimize false positivity, resulting in small group size in the IL-10-high group. Therefore, further optimization of the cut-off value and external validation are required in subsequent study. Seconds, we did not elucidate the immunological mechanisms whereby serum IL-10 levels affected ICI responses.

## Conclusions

High baseline serum IL-10 levels are associated with poor clinical outcomes in patient with advanced RCC treated with first-line ICI therapy. Further validation of pretreatment IL-10 levels is warranted to optimize first-line ICI therapy in patients with advanced RCC.

## Supplementary Material

Supplementary figure and table.

## Figures and Tables

**Table 1:** Clinical characteristic according to IL-10 status in RCC patients (cut-off = 4.3 ng/mL)

	Total (N=69)	IL-10-low (N=60)	IL-10-high (N=9)	P-value
**Regimen**				
pembrolizumab + axitinib	58 (84.1%)	49 (81.7%)	9 (100.0%)	0.505
ipilimumab + nivolumab	9 (13.0%)	9 (15.0%)	0 (0.0%)	
pembrolizumab + lenvatinib	2 (2.9%)	2 (3.3%)	0 (0.0%)	
**Age**	59 (19-83)	59 (19-82)	59 (47-83)	0.260
**Sex**				
Female	20 (29.0%)	17 (28.3%)	3 (33.3%)	0.712
Male	49 (71.0%)	43 (71.7%)	6 (66.7%)	
**Pathology**				
Clear cell	47 (68.1%)	40 (66.7%)	7 (77.8%)	0.708
Non-clear cell	22 (31.9%)	20 (33.3%)	2 (22.2%)	
**IMDC**				
Favorable	15 (21.7%)	15 (25.0%)	0 (0.0%)	0.094
Intermediate	34 (49.3%)	30 (50.0%)	4 (44.4%)	
Poor	20 (29.0%)	15 (25.0%)	5 (55.6%)	
**ECOG**				
0	27 (39.1%)	24 (40.0%)	3 (33.3%)	0.546
1	34 (49.3%)	30 (50.0%)	4 (44.4%)	
2	8 (11.6%)	6 (10.0%)	2 (22.2%)	
**Nephrectomy**				
No	30 (43.5%)	24 (40.0%)	6 (66.7%)	0.163
Yes	39 (56.5%)	36 (60.0%)	3 (33.3%)	
**Best response**				
Complete response (CR)	5 (7.2%)	5 (8.3%)	0 (0.0%)	0.862
Partial response (PR)	28 (40.6%)	23 (38.3%)	5 (55.6%)	
Stable disease (SD)	24 (34.8%)	21 (35.0%)	3 (33.3%)	
Progressive disease (PD)	12 (17.4%)	11 (18.3%)	1 (11.1%)	
