# Supplementary material for: High levels of baseline serum IL-10 are associated with reduced clinical benefit from first-line immune checkpoint inhibitor therapy in advanced renal cell carcinoma
Source: J Cancer. 2023 Apr 2;14(6):935–42. doi: 10.7150/jca.81384 (PMC10158513; doi:10.7150/jca.81384)

Supplementary Table 1. Response rate according to the C2D1/Baseline ratio

	Total (n=52)	No change or <1.5 (n=26)	1.5-2.0 fold increase (n=11)	≥2.0 fold increase (n=15)	P-value
CR	3(5.8%)	1(3.8%)	1(9.1%)	1(6.7%)	0.673
PR	26(50.0%)	14(53.8%)	6(54.5%)	6(40.0%)	
SD	19(36.5%)	10(38.5%)	3(27.3%)	6(40.0%)	
PD	4(7.7%)	1(3.8%)	1(9.1%)	2(13.3%)	

Supplementary figure 1. **Survival outcomes according to IL-10 changes.**

PFS (A) and OS (B) of the patient with advanced RCC according to the C2D1/Baseline: No change or <1.5 fold (n=26), 1.5-2.0 fold increase (n=11), and ≥2.0 fold increase (n=15). P-values were calculated using the log-rank test.

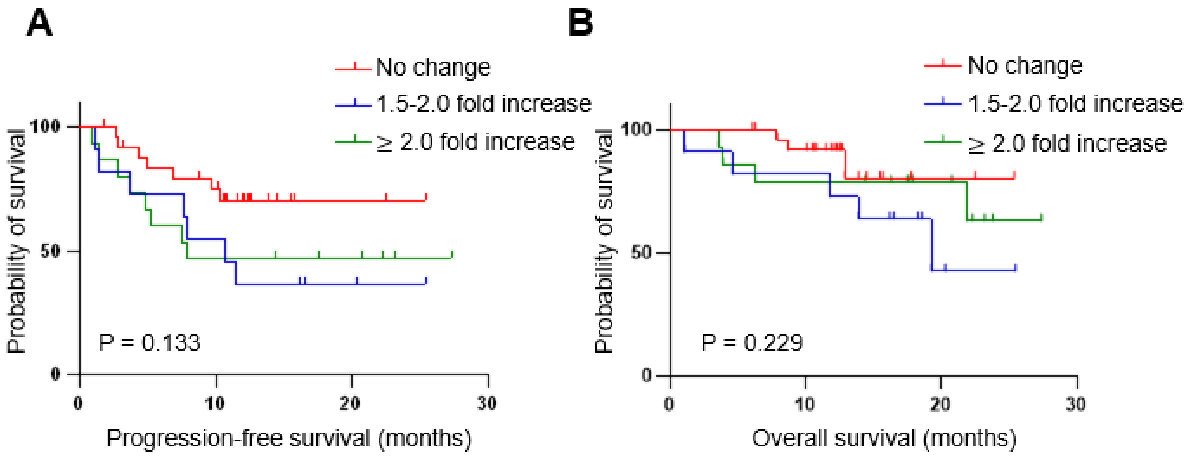

Supplement: Supplementary file 1 — Supplementary figure and table. [file jcav14p0935s1.pdf]
